# Anaesthesia personnels’ perspectives on digital anaesthesia information management systems – a qualitative study

**DOI:** 10.1186/s12912-022-00998-9

**Published:** 2022-08-01

**Authors:** Ann-Chatrin Leonardsen, Anne-Marie Gran Bruun, Berit T. Valeberg

**Affiliations:** 1 Østfold University College/Østfold Hospital Trust, Postal box code 700, 1757 Halden, Norway; 2 University of Southeastern Norway, Postal box code 235, 3603 Kongsberg, Norway; 3 Oslo Metropolitan University / University of Southeastern Norway, Pilestredet 32, 0166 Oslo, Norway

**Keywords:** Nurse anaesthetist, Anaesthetist, Digital AIMS, Implementation, Vigilance

## Abstract

**Background:**

In Norway, the anaesthesia team normally consists of a nurse anaesthetist and an anaesthetist. Digital anesthesia information management systems (AIMS) that collect patient information directly from the anaesthesia workstation, and transmit the data into documentation systems have recently been implemented in Norway. Earlier studies have indicated that implementation of digital AIMS impacts the clinical workflow patterns and distracts the anaesthesia providers. These studies have mainly had a quantitative design and focused on functionality, installation designs, benefits and challenges associated with implementing and using AIMS. Hence, the aim of this study was to qualitatively explore anaesthesia personnel’s perspectives on implementing and using digital AIMS.

**Methods:**

The study had an exploratory and descriptive design. The study was conducted within three non-university hospitals in Southern Norway. Qualitative, individual interviews with nurse anaesthetists (*n* = 9) and anaesthetists (*n* = 9) were conducted in the period September to December 2020. Data were analysed using qualitative content analysis according to the recommendations of Graneheim and Lundman.

**Results:**

Four categories were identified: 1) Balance between clinical assessment and monitoring, 2) Vigilance in relation to the patient, 3) The nurse-physician collaboration, and 4) Software issues. Participants described that anaesthesia included a continuous balance between clinical assessment and monitoring. They experienced that the digital AIMS had an impact on their vigilance in relation to the patient during anaesthesia. The digital AIMS affected the nurse-physician collaboration. Moreover, participants emphasised a lack of user participation and aspects of user-friendliness regarding the implementation of digital AIMS.

**Conclusion:**

Digital AIMS impacts vigilance in relation to the patient. Hence, collaboration and acceptance of the mutual responsibility between nurse anaesthetists and anaesthetists for both clinical observation and digital AIMS administration is essential. Anaesthesia personnel should be included in development and implementation processes to facilitate implementation.

## Introduction

A central part of nurse anaesthetists’ work is to monitor, observe, document and assess the depth of anaesthesia, as well as the effects of anaesthetics on patients’ ventilation and circulation. Nurse anaesthetists must also be aware of potential problems, and administer anaesthesia while continuously observing and assessing the patients’ clinical status [1]*.* Situation awareness is assumed an essential skill, relating to whether changes in patients’ condition are recognised, understood and foreseen [2, 3]. Patient and equipment monitoring is used to titrate administration of anaesthetic medication, to detect physiologic perturbations and allow intervention before the patient suffers harm, and to detect and correct equipment malfunction [4]. A 2021 study found that the highest task frequency for nurse anaesthetists was during anaesthesia induction, which has also been identified as a safety-critical phase [5].

Clinical monitoring is supplementary to monitoring devices and includes visual inspection, auscultation, and palpation [4, 6]. Moreover, clinical monitoring includes cognitive and behavioral skills to assess the context, the patients’ condition, the team or available equipment and medications [7–9].

Implementation of new digital solutions can lead to changes in the performance of anaasthesia. Patient safety issues have been suggested as a facilitator for such implementation [10]. Anesthesia information management system (AIMS) is a collective term for systems that collect patient information from the anesthesia workstation and transfer the data directly into documentation systems [11]. Earlier studies have focused on functionality, installation designs, benefits and challenges associated with implementing and using digital AIMS [12–14]. Moreover, studies indicate that implementation of digital AIMS leads to improved documentation and more accurate capture of perianaesthetic data [12–14]. Disadvantages include resistance to replacing paper records, unacceptable installation and maintenance costs, distraction of the anesthesia providers, and resistance to changes in clinical workflow patterns [15–17]. A 2021 literature review [18] identified only seven studies published in the period 1991–2018 focusing on anaesthesia personnels’ perspectives on digital AIMS, all of them with a quantitative approach. Results showed that the majority of anesthesia personnel were satisfied with the digital AIMS, even though stating issues related to technical aspects, physical placement of the equipment and quality of care, leading to suggestions for improvement.

## Background

In Norway, nurse anaesthetists are qualified to independently administer general anaesthesia for minor operations on otherwise healthy patients, provided that an anaesthetist has assessed the patient as fit for anaesthesia and can be called upon if needed. Moreover, nurse anaesthetists are qualified to work in a team with an anaesthetist in major surgery and patients with more complex illnesses, as well as to monitor patients during regional anaesthesia, sedation and general anaesthesia. The anaesthesia team normally consists of an anaesthetist and a nurse anaesthetist. The anaesthetist may be responsible for several patients under anaesthesia at the same time, hence the nurse anaesthetists are present continously during anaesthesia [19]. The Norwegian standard for the safe practice of anaesthesia [19] states that vital signs must be documented regularly, depending on the patient’s condition and the complexity of the intervention. Moreover, Norwegian healthcare personnel are legislated to document ‘relevant and neccessary information’ about the patient and treatment [20].

Betza et al. [21] found that there was a high frequency of task transitions to looking at the visual displays and then from the visual displays towards the patient, indicating that technology impacts vigilance on the patient. In contrast, Tse et al. [22] compared AIMS and manual record keeping, and found no significant difference in anaesthesia personnels’ vigilance detection accuracy. However, the result for situation awareness accuracy was inconclusive as the study did not have enough power to detect a difference between the two conditions. Beyond these two studies, we have not been able to identify studies analysing the effects of AIMS on comprehensive monitoring performance.

Digital AIMS have recently been implemented in Norwegian hospitals, as a system called Metavision®. Simply providing healthcare professionals with new technology is unlikely to lead to the transformation in health care that the new technology is proposed to deliver [23]. It is widely recognised that interventions are most effective when based on behaviour change theory and techniques [24, 25]. Earlier studies have mainly focused on the practical use of AIMS rather than how this is implemented and utilised alongside clinical monitoring [26–28]. Moreover, previous studies on digital AIMS have taken a quantitative approach. Qualitative studies are appropriate for in-depth exploration of people’s experiences and perspectives [29]. The rationale of this study was to assess whether issues related to technical aspects, physical placement of the equipment, and distraction due to the AIMS as stated in previous studies [15–17] were present in a Norwegian setting, and whether these issues were experienced as a risk to peroperative patient safety or quality of anaesthesia care. Hence, the aim of this study was to explore anaesthesia personnels’ perspectives on the implementation of AIMS using qualitative interviews.

## Materials and methods

The study had a qualitative design and included individual interviews with anaesthesia personnel in the period September to December 2020. The researchers were all female nurse anaesthetists, two of whom had a PhD and all experienced with qualitative interviewing. The study adheres to the Consolidated Criteria for Reporting Qualitative Research (COREQ) [30].

### Setting and participants

The study was conducted in three hospitals in Southern Norway. We used a purposive sampling strategy, to identify information-rich cases related to the phenomenon of interest [31]. Managers from the anaesthesia department in the three hospitals were asked to invite three nurse anaesthetists and three anaesthetists respectively to participate. Inclusion criteria were a minimum 50% clinical work and having worked in the anaesthesia department during the past year. Managers were asked to select candidates with a variation in gender, age and years of anaesthesia experience.

### Data collection

An interview guide was developed based on current research on AIMS (e.g. [12, 13, 32, 33]) and several discussions between the researchers (see Table 1). The guide was piloted in two experienced nurse anaesthetists. The pilot lead to minor changes in the interview guide, and the questions were deemed relevant and understandable.

**Table 1:** Interview guide

Questions	Follow ups
1. Has clinical monitoring changed throughout your career?	If so, in what way? Do you think this change has been to the better or to the worse?
2. How do you monitor the patient during anaesthesia?	In what way do you gather the observations (technology/clinical observation)
3. How do you document the clinical observations?	Do you document continously when something occurs, or in retrospect?
4. Who do you think is responsible for documentation?	
5. Are there any differences between short and long procedures regarding clinical observations and documentation?	
6. Are there any differences between acute and elective procedures regarding clinical observations and documentation?	
7. Are there any differences between general- and regional anaesthesia regarding clinical observations and documentation?	
8. Are there any advantages with digital AIMS in short procedures?	What about in long procedures?
9. Are there any disadvantages with digital AIMS in short procedures?	What about in long procedures?
10. How do you think digital AIMS positively affects patient safety?	
11. How do you think digital AIMS negatively affects patient safety?	
12. How do you collaborate within the anaesthesia team regarding clinical monitoring, documentation and use of digital AIMS?	
13. Is there anything else you would like to add about digital AIMS, that we have not asked you?	

The researchers interviewed three nurse anaesthetists and three anaesthetists from one hospital each. Twelve of the interviews were conducted in a meeting room near the anaesthesia department in the hospitals. Due to the COVID-19 pandemic, six interviews were conducted digitally on a secure platform (Teams^R^) with both sound and video. This method has been widely used and is a viable alternative to face-to-face interaction [34]. The audio of the interviews was digitally recorded and transcribed verbatim by an external transcriber, who had signed a non- disclosure agreement. The recordings were deleted after transcription. Saturation was reached after the 18 interviews, meaning that no new themes were identified [35].

### Analysis

From a critical realist point of view, the world as we know and understand it is constructed from our perspectives and experiences, through what is ‘observable’ [36]. Content analysis makes sense of what is mediated between people including textual matter, symbols, messages, information, mass-media content, and technology supported social interactions [37]. Hence, we used inductive qualitative content analysis according to the recommendations of Graneheim and Lundman, [38] following a five-step procedure. The interviews were read through several times to obtain a sense of the whole. In step one, we identified meaning units: words, sentences or paragraphs containing aspects related to each other through their content and context within each transcript. This step was done by each researcher respectively, and then compared and discussed until consensus was reached. In step two, meaning units were shortened while still preserving the core, leading to condensed meaning units. In the third step, the condensed meaning units were abstracted, including an interpretation of the underlying meaning. The condensed meaning units were then labelled with a code in the fourth step. In step five, the various codes were compared across all interviews based on differences and similarities, and sorted into categories. Steps two to five were conducted in physical meetings with all authors present and discussed until consensus was reached.

In addition, reflexivity was used as a method [39]. Reflexivity notes including the researchers’ preconceptions, thoughts and ideas were written down before each interview. After each interview, notes were taken regarding interview dynamics and behaviour of the interviewer and the participant that could potentially impact the analysis. These notes were included in the interpretation and discussions throughout the analytic process.

### Ethics

The study was conducted in line with the ethical guidelines for research in the Declaration of Helsinki [40]. All participants provided willing, informed, written consent to participate. The study was approved by the Norwegian Centre for Research Data (NSD) (project no. 599254), as well as the privacy representative in each participating hospital respectively. According to Norwegian legislation, no ethical approval was needed.

## Results

A total of nine nurse anaesthetists (five female) and nine anaesthetists (four female) participated in the study. The age range was 30 to 58 years (mean 42.8) and years of anaesthesia experience ranged from 1 to 23 years (mean 9.2). Through analysis we identified four categories: 1) Balance between clinical assessment and monitoring, 2) Vigilance in relation to the patient, 3) The nurse-physician collaboration, and 4) Software issues.

### Balance between clinical assessment and monitoring

All of the participants reflected on how they balanced clinical assessment of their patients and the information the digital AIMS provided. They also described their continuous clinical assessments during anaesthesia. In addition, several of the participants pointed out the challenges that arose when the clinical observation did not match the automatic measurement. This made some of them feel insecure, even though most of them then interpreted the measurements as errors and started searching for equipment failure. Participant 11, an anaesthetist with 4 years of anaesthesia practice reported,

*“You have to decide what is actually going on with the patient, and which actions should I make now … And the answer is often to look at the measurement … where’s the deviation, and what causes this deviation. The answer is often disturbances related to the surgical procedure, and maybe not in one isolated measurement”*.

The digital AIMS was introduced to improve documentation and registration. Nevertheless, participant 19, an experienced nurse anaesthetist claimed that “*it [the AIMS] is not a tool to document clinical assessments*”. Participant 18, an anaesthetist with 6 years of anaesthesia practice, stated,

“*The AIMS is just electronic. What you think and feel is not registered … The physical signs, like clammy skin, sweat, anxiety … Things that are not automatically registered. You’ll have to write … But then again, this was not documented in paper either*”.

All of the participants stated that they responded to and acted on clinical signs such as clammy, pale skin, even if this was not documented.

### Vigilance in relation to the patient

All of the participants described that the digital AIMS had an impact on their vigilance in relation to the patient in one way or another. Still, there was no consensus between participants, or even within individual participants’ interviews, on whether the impact was positive or negative. Ten of the participants believed that the digital AIMS reduced vigilance because it took the focus off the patients. Participant 1, a female anaesthetist, stated that “*the screen*” lead to nurses being “*tied up*”, being “*too busy with typing*”. Several of the other participants agreed with this, especially in relation to induction and emergence of anaesthesia. Participants 2, 15, 16 and 18 also stated that system errors also “*stole focus*” from the patient.

Four participants (participants 1, 10, 13 and 16), all with more than 8 years of anaesthesia practice and one of them a nurse anaesthetist, claimed that the AIMS increased the focus on the patient while manual documentation took the focus off of the patient. Participant 13 elaborated:

“*We write paper curves during traumas, and I recognize how much focus it takes to write down the values; it takes almost one person in large teams. Hence, I felt it was a burden to write paper curves compared to automatic systems*”.

But still, this female nurse anaesthetist also described disadvantages related to the AIMS:

“*In short procedures, it feels like you only get to punch in the data before you have to end, and then you risk losing focus on the patient, the clinical observation, due to all the typing. And it may also be a disadvantage when leaving the operating room … [It takes] many keystrokes to transfer the curve and end the anaesthesia*”.

Moreover, participant 16, a male anaesthetist, perceived that the AIMS “*freed up time*”. Still, he also described negative effects of the AIMS: “*There are so many things to document, and then the documentation itself becomes most important*.”

### The nurse-physician collaboration

All of the participants had various perspectives on the collaboration between the nurse anaesthetist and the anaesthetist related to the AIMS. Most of the participants thought that the AIMS was most often administered by the nurse anaesthetists. In contrast, Participant 1, an experienced anaesthetist, claimed that documentation was her responsibility, although she also confirmed that the nurse anaesthetists “*kept the overview*”. Moreover, she reported that the anaesthetists always documented special incidents or adverse events.

Participant 16, another experienced anaesthetist supported this, adding that.

“*procedures and descriptions are also the anaesthetists’ responsibility*.”

Even though several of the participants agreed that the AIMS was a joint responsibility, most of them accepted that it was the nurse anaesthetists who assumed it. This distribution of tasks, which was reported by several participants, lead to the anaesthetist taking care of the patient while the nurse anaesthetist handled the AIMS. In many situations, induction of patients required additional personnel because the responsible nurse anaesthetist was fully occupied with “*the system*”. Otherwise, this would lead to a delay in recording, which would in turn lead to a higher workload for the nurse anaesthetist during surgery since the anaesthetists most often left the room directly after the induction. Participant 4, an experienced nurse anaesthetist, even claimed that “*only nurse anaesthetists add both clinical actions and documentation*”. Participant 18, an anaesthetist with 6 years of experience, had another point of view. He stated,

“*I think it has become a trend, that the physicians become more practically active, and that the nurses unfortunately are standing more on the side … And this leads to that I, unfortunately, experience that some of the nurse anesthetists do not have the same practical skills as before*”.

Participant 12, an experienced anaesthetist, reported another advantage: namely that the AIMS made it possible to monitor the anesthesia from a distance, enabling the anaesthetist to monitor several operating rooms at a time. Hence, the introduction of the AIMS was reported to change the collaboration and the distribution of tasks between anaesthesia personnel.

### Software issues

Four of the participants, two nurse anaesthetists and two anaesthetists, talked about the lack of user participation in the development, introduction and implementation of the AIMS. Participant 11, an unexperienced anaesthetist, commented, “*It’s developed in Israel or Canada … I don’t feel that anyone in this hospital is allowed to give input … And there has to be an enormous consensus that a function is not optimal*”.

Participant 7, an experienced nurse anaesthetist, added, “… *it was introduced almost without us asking for it, and then we have to relate to it …*”.

Eight of the participants also talked about “user friendliness” in relation to the digital AIMS in negative terms. The digital AIMS was reported to be “*cumbersome*”, “*time-consuming*”, and creating an “*information overload*”. Three of the participants emphasised that the important information was not separated from unimportant information and that it was difficult to identify necessary information. The main problem seemed to be that the AIMS was not specifically designed for the anesthesia team but was developed for use in all hospital departments. Nevertheless, the participants also accepted that it had advantages. For example, Participant 15, an experienced nurse anaesthetist, shared the following reflection:

“*Drug administration is very tidy in the AIMS, and you get a clear overview of what is done. I have a bad handwriting which may lead to misinterpretations. I like the automatic aspect, that all vital parameters are transferred*”.

Moreover, all of the participants reported that the AIMS was source of several errors. If the patient was not transferred into the system, the AIMS could not be used, and the automatic data collection was then delayed. Several areas that needed improvement were also highlighted, such as the fluid account, the possibility to end the registration in the postoperative anesthesia care unit (PACU), and avoidance of double registration, which was now quite common. Participant 2, an experienced nurse anaesthetist, sighed and explained,

“*You have to connect the patient to the equipment before induction. And then you have to check that the monitor is working and that the parameters are gathered. And at the same time, you have to register in another system, because they do not talk to each other*”.

The digital AIMS was also “down” quite often, leading to a need to go back to the old paper-based documentation system. This again led to challenges because inexperienced anaesthesia personnel were not trained to use the paper-based system.

Ten of the participants reported that the digital AIMS had increased the trustworthiness of anaesthesia documentation. This was related to measurements being automatically collected in real time. Several of the participants reported that the digital AIMS was more precise than handwritten records. Three of the participants even reported that in the past, it was possible to “embellish” records. For example, participant 10, an experienced anaesthetist, said,

“*The experience before automatic gathering was that, in acute situations, we either did not manage to record what happened, and we did not know what the lowest blood pressure was. And we did not know when the cardiac arrest occurred because we were busy with the patient. And then, the tendency to embellish the picture … to set a higher pressure than it really was … And now, we’re not allowed to cheat, and that’s a good thing*”.

But even though participants accepted that the digital AIMS led to a more accurate picture of the anaesthesia, they did not find that this had increased patient safety.

## Discussion

When describing anaesthesia, participants in our study cited a continuous balance between clinical assessment and monitoring. They perceived that the digital AIMS had an impact on their vigilance during anaesthesia, when compared to traditional paper records. The digital AIMS also had an impact on the nurse-physician collaboration. When the nurses took most of the responsibility for the digital AIMS documentation, the physician could focus on the patient. Moreover, participants emphasised lack of user participation in the development and implementation of the digital AIMS and aspects of user friendliness.

All of the participants described their continuous clinical assessments during anaesthesia and noted that this was their basis for decision-making. Hence, the digital AIMS was seen as a supplement to balanced anaesthesia. This approach has been supported in several other studies [4, 6, 41]. Only four of the participants claimed that the old paper-based documentation system took the focus off of the patient, whereas most of the participants claimed that the digital AIMS decreased vigilance, especially in relation to induction and emergence of anaesthesia. This contrast also appears in earlier studies, which have indicated both a resistance to abandoning paper records and distraction among anesthesia providers due to implementation of digital AIMS [15–17]. The time consuming nature of data entry is also supported in earlier studies, as well as difficulties learning the system [16, 33].

The implementation of the digital AIMS was perceived to change the collaboration between nurse anaesthetists and anaesthetists, leading to nurses being more occupied with the digital AIMS, especially during induction and emergence of anaesthesia. Expertise in anaesthesia requires regular practice, continuing professional development and annual training on acute interventions, including communication and teamwork [19]. A change in the distribution of tasks may lead to nurse anaesthetists’ not getting regular practice in induction techniques such as drug administration or securing airways under induction. In Norway, nurse anaesthetists are most commonly the ones present throughout anaesthesia, while anaesthetists may be responsible for several patients under anaesthesia at the same time [19]. This distribution of tasks has also been described internationally [42, 43]. According the American Society of Anesthesiologists, documentation is a basic responsibility of physician anaesthetists [44]. Participants in our study accepted this as a mutual responsibility. To ensure anaesthesia training for both nurse anaesthetists and anaesthetists, we suggest that all anaesthesia personnel participate equally in documentation and that anaesthetists get more supervision and training in using the digital AIMS.

Anaesthesia personnel reflected on user participation, user-friendliness and trustworthiness in relation to implementation of the digital AIMS. Several of the participants described that the digital AIMS had been developed and implemented without input from anaesthesia personnel, and without them expressing a need for change. A systematic review showed that facilitators of implementation processes in hospitals were motivation to change, personal beliefs regarding the intervention, understanding of end goals and outcomes, and level of skill and confidence among healthcare personnel [45]. When this is lacking, as indicated in our study, implementation may not succeed. In addition, several of the participants found the digital AIMS to not be user friendly. This is in contrast with findings in earlier studies [16, 32, 33, 46, 47].

The purpose of implementing the digital AIMS was assumed to be improved patient safety. Participants in our study reported that the digital AIMS provided more trustworthy documentation, but that this did not necessarily correspond to increased patient safety. Earlier studies indicate that implementation of digital AIMS leads to improved documentation and more accurate capture of perianaesthetic data [12–14]. This was supported by findings in our study. However, it could be claimed that the digital AIMS also negatively impact patient safety, due to being time consuming, and impacting vigilance on the patient as indicated by our findings. Patient safety in the operating room is understood as the responsibility of all professionals working in it [48]. Hence, it could be argued that other personnel than the anaesthesia personnel should take part in digital AIMS completion or patient montitoring, especially under induction or emergence of anaesthesia.

Digital AIMS has come to stay, and anaesthesia personnel have to cope with this technology [14]. AIMS-based clinical decision support systems can significantly improve some aspects of clinical performance and patient care, particularly if the decision support is smoothly integrated into clinical workflow [49]. As such, multitasking, or managing multiple tasks simultaneously^,^ has been described as an integral and appropriate part of acute care [50–52]. Multitasking and interruptions being seen as threats to safety in anaesthesia care, could be interpreted as signs of the adaptive capacity of a complex system, reflecting resilience [5]. Our findings may inform developers and users of digital AIMS about concerns they should consider during development and implementation to increase effectiveness and mitigate potentially disruptive aspects of this technology.

Even though our study was conducted in anaesthesia personnel, our findings may be transferable to other healthcare personnel and healthcare settings. Digital information management systems such as Metavision® are implemented in hospitals internationally. How such implementation impacts the balance between clinical assessment and monitoring, the vigilance in relation to the patient, or the collaboration between healthcare personnel may be similar in other hospital wards than anaesthesia. Moreover, software issues and the importance of including stakeholders when developing and implementing new systems and/or technology are highly relevant across healthcare settings.

### Methodological considerations

This is, to our knowledge, the first qualitative study exploring anaesthesia personnels’ perspectives on digital AIMS. The study was conducted in a Norwegian setting; hence, findings may not be generalisable to all settings. Nevertheless, the transferability of our findings is supported by the inclusion of both nurse anaesthetists and anaesthetists from three different hospitals with variation in gender, age and years of anaesthesia experience.

Credibility and trustworthiness were achieved through transparency in the thorough description of the data collection and analysis, the use of reflexivity, as well as the presentation of illustrative quotes. Rigour was ensured through a systematic approach throughout the study, as well as iterative discussions within the research group.

The researchers were all nurse anaesthetists. Including an anaesthetist (male) in the research team might have affected both what the participants expressed and the interpretation of our findings. Our impression is that the participants were open and gave rich descriptions of their perspectives regardless of gender or professional background.

Moreover, we could have included an anaesthetist (male) when piloting the interview guide. However, we finished all the interviews asking whether the participants had something to add beyond what we asked. Here, only one participant, a male anaesthetist, added that he would never go back to paper-based AIMS again. This indicate that our questions covered all relevant aspects of digital AIMS in both nurse anaesthetists and anaesthetists. The validity of our findings could also been improved through letting participants read through and give feed-back on the transcripts and analysis of findings. This was not done.

## Conclusion

This study provides in-depth knowledge about anaesthesia personnels’ perspectives on digital AIMS. Findings indicate that digital AIMS impacts vigilance. It is important to achieve close collaboration between nurse anaesthetists and anaesthetists, and acceptance of a mutual responsibility for both clinical observation and digital AIMS administration. Personnel need to understand the purpose and outcomes of an innovation, which was not necessarily the case with the implementation of the digital AIMS. Hence, it is essential to include anaesthesia personnel in the implementation processes of new technology to facilitate implementation.

### Implications

Digital AIMS is time-consuming, especially at induction and emergence of anaesthesia. This aspect is essential to resource planning in operating theatres to ensure patient safety.

## Data Availability

The datasets used and/or analysed are available from the corresponding author on reasonable request.

## References

[1] Nilsson U, Jaensson M (2016). Anesthetic nursing: keep in touch, watch over, and be one step ahead. J Peranesth Nurs.

[2] Schultz C, Krautheim V, Hackemann A. Situation awareness errors in anesthesia and critical care in 200 cases of a critical incident reporting system. BMC Anesthesiol. 2016;16(4). 10.1186/s12871-016-0172-7. 10.1186/s12871-016-0172-7 PMC4715310 26772179

[3] Flin R, O'Connor P, Crichton M (2008). Safety at the sharp end: a guide to non-technical skills.

[4] Iohom G. Monitoring during anesthesia. UpToDate2019 [updated Apr 18 2019. Available from: https://www.uptodate.com/contents/monitoring-during-anesthesia (Accessed 02/02/2022).

[5] Olin K, Göras C, Nilsson U, Unbeck M, Ehrenberg A, Pukk-Härenstram K, et al. Mapping registered nurse anaesthetists' intraoperative work: tasks, multitasking, interruptions and their causes, and interactions: a prospective observational study. BMJ Open. 2021;12(e052283). 10.1136/bmjopen-2021-052283. 10.1136/bmjopen-2021-052283 PMC8772415 35045998

[6] Szostakiewicz K, Rybicki Z, Tomaszewski D (2018). Non-instrumental clinical monitoring does not guarantee an adequate course of general anesthesia. A prospective clinical study. Biomed Pap med Fac Univ Palacky Olomouc Repub.

[7] Keating-Biltucci M, Majeed Z, Zollo R, Ward D. Combining anesthesia non-technical skills and peer-learning in the operating room. MedEdPublish. 2017. 10.15694/mep.2017.000097.

[8] Flin R, Patey R, Glavin R, Maran N (2010). Anaesthetists’ non-technical skills. BJA.

[9] Lyk-Jensen H, Jepsen R, Spanager L, Dieckmann P, Østergaard D (2014). Assessing nurse anaesthetists' non-technical skills in the operating room. Acta Anaesthesiol Scand.

[10] Dugstad J, Sundling V, Nilsen E (2020). Nursing staff's evaluation of facilitators and barriers during implementation of wireless nurse call systems in residental care facilities. A cross-sectional study. BMC Health Serv Res.

[11] Peterson J, White K, Westra B, Monsen K (2014). Anesthesia information management systems: imperatives for nurse anesthetists. AANA J.

[12] Kadry B, Feaster W, MacArio A, Ehrenfeld J (2012). Anesthesia information management systems: past, present, and future anesthesia records. Mount Sinai J Med.

[13] Ehrenfeld J, Rehman M (2011). Anesthesia information management systems: a review of functionality and installation considerations. J Clin Monit Comput.

[14] Simpao A, Rehman M (2018). Anesthesia information management systems. Anesth Analg.

[15] Gardner R, Pralash O (1994). Challenges and opportunities for computerizing the anesthesia record. J Clin Anesth.

[16] Quinzio L, Junger A, Gottwald B (2003). User acceptance of an anaesthesia information management system. Eur J Anaesthesiol.

[17] Bloomfield E, Feinglass N (2008). The anesthesia information management system for electronic documentation: what are we waiting for?. J Ameth.

[18] Leonardsen A, Gran Bruun A, Valeberg B (2021). Anesthesia personnels' experiences with digital anesthesia information management systems: a literature review. AANA J..

[19] Ringvold E, Bekkevold M, Bruun A, Børke W, Finjarn T, Haugen A (2018). Norwegian standard for the safe practice of anaesthesia. Acta Anaesthesiol Scand.

[20] Norwegian Department of Health and Care. Legislation on healthcare personnel. 2022.§§39–40. Available from: https://lovdata.no/dokument/NL/lov/1999-07-02-64#KAPITTEL_8 (accessed June 20 2022).

[21] Betza S, Jurewicz K, Neyens D, Riggs S, Abernathy J, Reeves S. Anesthesia maintenance and vigilance: examining task switching. Proc Human Factors Ergon Soc. 2016:608–12.

[22] Tse M, Li S, Chiu T, Lau C, Lam K, Cheng C (2020). Comparison of the effects of automated and manual record keeping on anesthetists' monitoring performance: randomized controlled simulation study. JMIR Hum Factors.

[23] Keyworth C, Hart J, Armitage C, Tully M (2018). What miximizes the effectiveness and implementation of technology-based interventions to support healthcare professional practice? A systematic literature review. BMC Med Inform Decision Making.

[24] Gardner B, Whittington C, McAteer J, Eccles M, Michie S (2010). Using theory to synthesise evidence from behaviour change interventions: the example of audit and feedback. Sco Sci Med.

[25] Michie S, Richardson M, Johnston M, Abraham C, Francis J, Hardeman W (2013). The behavior change technique taxonomy (v1) of 93 hierarchically clustered techniques: building an international consensus for the reporting of behavior change interventions. Ann Behav Med.

[26] Rozental O, White R (2019). Anesthesia information management systems: evolution of the paper anesthetic record to a multisystem electronic medical record network that streamlines perioperative care. J Anesth Hist.

[27] Wilbanks B, Berner E, Alexander G, Azuero A, Patrician P, Moss J (2018). The effect of data-entry template desgin and anesthesia provider workload on documentation accuracy, documentation efficiency and user-satisfaction. In J Med Inform.

[28] Pysyk C, Jee R, Zunder I (2019). Change in staff anesthesiologists' opinions of an Anestheisa information management system (AIMS). J Clin Monit Comput.

[29] Braun V, Clarke V (2013). Successful qualitative research: a practical guide for beginners.

[30] Tong A, Sainsbury P, Craig J (2007). Consolidated criteria for reporting of qualitative research (COREQ): a 32-item checklist for interviews nd focus groups. Int J Qual Health Care.

[31] Palinkas L, Horwitz S, Hoagwood K (2015). Purposeful sampling for qualitative data collection and analysis in mixed method implementation research. Adm Policy Ment Health.

[32] DeVos C, Abel M, Abenstein J (1991). An evaluation of an automated anesthesia record keeping system. Biomed Sci Instrum.

[33] Abenstein J, DeVos C, Tarhan S (1992). Eight year’s experience with automated anesthesia record keeping: lessons learned- new directions taken. Int J Clin Monit Comput.

[34] Iacono V, Symonds P, Brown D. Skype as a tool for qualitative research interviews. Sociol Res Online. 2016;21(2). 10.5153/sro.3952.

[35] Guest G, Namey E, Chen M (2020). A simple method to asses and report thematic saturation in qualitative research. PLoS One.

[36] Gorski P (2013). What is critical realism? And why should you care?. Contemp Soc J Rev.

[37] Vaismoradi M, Turunen H, Bondas T (2013). Content analysis and thematic analysis: implications for cinducting a qualitative descriptive study. Nurs Health Sci.

[38] Graneheim U, Lundman B (2004). Qualitative content analysis in nursing research: concepts, procedures and measures to achieve trustworthiness. Nurse Educ Today.

[39] Del Busso A, Leonardsen A (2020). Qualitative research as reflexive process: a word limitation challenge in qualitative medical research publications?. Nordic Nurs Res.

[40] World Medical Association. Declaration of Helsinki- Ethical Principles for Medical Research Involving Human Subjects 2015. Available from: http://www.wma.net/en/30publications/10policies/b3 (Accessed 02/02/2022).

[41] Schreiber R, MacDonald M (2010). Keeping vigil over tha patient: a grounded theory of nurse anaesthesia practice. JAN.

[42] Herion C, Egger L, Greif L, Violato C (2019). Validating international CanMeds-based standards defining education and safe practice of nurse anesthetists. Int Nurs Rev.

[43] International Council of Nurses. Guidelines on advanced practice nursing- nurse anesthetists. Geneva, Switzerland; 2021. Available from: https://www.icn.ch/system/files/2021-07/ICN_Nurse-Anaesthetist-Report_EN_WEB.pdf (Accessed 02/02/2022)

[44] American Society of Anesthesiologists (2018). Statement on documentation of anesthesia care: Committee on Quality Management and Departmental Administration.

[45] Geerligs L, Rankin N, Sheperd H, Butow P. Hospital-based interventions: a systematic review of staff-reported barriers and facilitators to implementation processes. Implement Sci. 2018;13(36). 10.1186/s13012-018-0726-9. 10.1186/s13012-018-0726-9 PMC5824580 29475440

[46] Beilin Y, Wax D, Torrillo T, Mungall D, Guinn N, Henriquez J (2009). A survey of anesthesiologists' and nurses' attitudes towards the implementation of an anesthesia information management system on a labor and delivery floor. Int J Obstetr Anesth.

[47] Jin H, Kim M, Lee S, Jeong H, Choi S, Lee H (2012). A survey of user acceptance of electronic patient anesthesia records. Korean J Anesthesiol.

[48] Fernandes A, Fassarella C, Camerini F, Henrique D, Nepomuceno R, Silva R (2021). Safety culture in the opertaing room: an integrative review. Rev Eletr Enferm.

[49] Nair B, Gabel E, Hofer I, Schwid H, Cannesson M (2017). Intraoperative clinical decision support for anesthesia: a narrative review of available systems. Anesth Analg.

[50] Douglas H, Raban M, Walter S (2017). Improving our understanding of multi-tasking in healthcare: drawing together the cognitive psychology and healthcare literature. Appl Ergon.

[51] Westbrook J, Raban M, Walter S. Task errors by emergency physicians are associated with interruptions, multitasking, fatigue and working memory capacity: a prospective, direct observation study. BMJ Qual Saf. 2018:655–63. 10.1136/bmjqs-2017-007333 PMC6204927 29317463

[52] Göras C, Olin K, Unbeck M (2019). Tasks, multitasking and interruptions among the surgical team in an operating room: a prospective observational study. BMJ Open.

